# Longitudinal Monitoring of Systemic Cytokines After Mild Zika Virus Infection Revealed an Association Between Th17 Polarization and Clinical and Serological Outcomes

**DOI:** 10.1002/jmv.70813

**Published:** 2026-01-20

**Authors:** Solène Marquine, Marie Mura, Franck de Laval, Gilda Grard, Cyril Badaut, Sébastien Briolant, Aurélie Trignol

**Affiliations:** ^1^ Unité de Virologie, Institut de Recherche Biomédicale des Armées Marseille France; ^2^ Unité des Virus Émergents (UVE: Aix‐Marseille Univ, Università di Corsica, IRD 190, Inserm 1207, IRBA) Marseille France; ^3^ Unité Interactions Hôte‐Pathogène, Institut de Recherche Biomédicale des Armées Brétigny‐sur‐Orge France; ^4^ Institut Pasteur, Interactomique, ARN et immunité Paris France; ^5^ Ecole du Val‐de‐Grâce Paris France; ^6^ Centre d'épidémiologie et de santé publique des armées Marseille France; ^7^ National Reference Center for Arboviruses, National Institute of Health and Medical Research (Inserm) and French Armed Forces Biomedical Research Institute (IRBA) Marseille France; ^8^ Unité de Parasitologie et Entomologie, Institut de Recherche Biomédicale des Armées Marseille France; ^9^ Aix‐Marseille Université, SSA, APHM, RITMES Marseille France; ^10^ IHU Méditerranée Infection Marseille France

**Keywords:** arbovirus, cytokines, neurological disorders, Th17 polarization, Zika virus

## Abstract

Zika virus (ZIKV) is a neurotropic virus that can cause a variety of neurological manifestations, ranging from mild forms to severe disorders like Guillain–Barré syndrome and congenital Zika syndrome. The pathophysiology of these complications is not fully understood, but they have been linked to host immune responses, particularly a proinflammatory Th1/Th17 profile. In this study, the kinetics of 14 cytokines were characterized in ZIKV‐infected patients recruited in French Guiana in 2016–2017. Cytokine concentrations were quantified using a multiplexed bead‐based immunoassay in serum samples collected sequentially from 36 patients during the first month after symptom onset. This longitudinal follow‐up provides chronological information on the immune response to mild‐to‐moderate ZIKV infection, with an early antiviral response dominated by IFN‐γ, TNF‐α and regulated by IL‐10, followed by a peak of Th1 and then Th17‐associated cytokines that persists for up to 1 month. The early presence of IL‐17A, IL‐21, and IL‐23 was positively correlated with the maximum amplitude of the serological response (total anti‐ZIKV IgG and seroneutralization titers), but also with the duration of neurological symptoms (paresthesia and muscle strength decrease), highlighting the bivalent role of Th17 immune response in ZIKV pathogenesis.

## 1 Introduction

The 2015–2017 epidemic in Central and South America brought global attention to the Zika virus (ZIKV). In most cases, ZIKV infection in humans leads to mild symptoms such as rash, pruritus, fever, arthralgia, and conjunctivitis [1]. However, various neurological and cardiovascular complications may occur [2, 3, 4]. In adults, manifestations, such as myocarditis, pericarditis, heart failure, and arrhythmias have been reported [4]. ZIKV is primarily associated with peripheral nervous system diseases [5] such as Guillain–Barré syndrome [6], which appears to affect men more frequently [7]. When maternal infection occurs during pregnancy, this neurotropic and cardiotropic virus can severely impair fetal brain development and is also associated with cardiac septal defects [8], heart failure [9], and myocarditis [10], leading to congenital Zika syndrome and microcephaly, which were reported at high frequency during the Latin American outbreak [11]. The severe neurological disorders have been associated with specific immune responses [12] such as elevated levels of CXCL‐10 (also termed IP‐10) or Th17 cytokines [13] but further characterization is required. ZIKV transmission occurs mainly through bites of *Aedes* mosquitoes, but also via sexual contact, blood transfusion, and vertical transmission [14]. The infection triggers local inflammation at the inoculation site, where innate immune cells secrete type I/II interferons and pro‐inflammatory cytokines, thereby restricting viral replication and initiating the adaptive response [15, 16]. Strong activation of T lymphocytes has been reported [17], with early IFN‐γ production by circulating CD8^+^ [18] and CD4^+^ T cells known to orchestrate the immune response with different T helper (Th) profiles, as well as a contribution of natural killer (NK) cells that eliminate infected targets in the early stages of the disease [19] in cooperation with cytotoxic T lymphocytes. Despite these observations, detailed analyses of immune cell populations during ZIKV infection remain limited. Most studies have assessed immune responses by measuring the levels of cytokines, chemokines or growth factors in the serum [20] or cerebrospinal fluids [21] of ZIKV‐infected patients. Nonsevere infections have been associated with Th1, Th2, Th17, and Th9 polarization [22, 23], but the reported responses vary depending on the immune mediators analyzed, the timing of sample collection, and cohort characteristics such as age, sex distribution (with a majority of women), and geographic origin (South of America [24] and Asia [23, 25]). Coinfections with other arboviruses, frequent in endemic areas, further complicate interpretation [26, 27]. Consequently, additional studies are required to better define the kinetics of ZIKV‐induced immune responses and their contribution to disease severity.

The clinical observations and serological results of a year‐long longitudinal follow‐up of a cohort of ZIKV‐infected patients recruited in French Guiana (ZIFAG cohort), between February 2016 and November 2017, have already been described [1, 28]. The most common symptoms were itchy skin rashes, asthenia and/or headaches, but paresthesia, reduced muscle strength, and areflexia were also transiently reported [1]. In the present work, the aim was to study the kinetics of 14 circulating cytokines in the serum of 36 patients with mild to moderate symptoms following a ZIKV infection, up to 1 month after symptom onset, in order to explore the polarization of the immune response and its association with clinical and serological outcomes.

## 2 Material and Methods

### 2.1 Study Design

Patients in the ZIFAG cohort attended 12 medical consultations spread over 1 year after the onset of symptoms; a precise description of the intensity and duration of all their symptoms was carried out in the first month, along with a standardized clinical examination, and venous blood samples were also taken [1]. The present cytokine study focused solely on the first 2–6 serum samples taken from 36 of these patients (all of them have been vaccinated against yellow fever, sex‐ratio: 2, median age: 39 years, 95% CI [35–42], min: 26, max: 63), that is, between Days 0 and 28 after symptom onset. The presence of ZIKV was confirmed in these samples by RT‐PCR [1]. Serum viral load has already been measured and described in Matheus et al. [29]. The control group comprised 67 ZIKV‐uninfected males (all of them have been vaccinated against yellow fever, median age: 31 years, 95% CI [27–33], min: 19, max: 48), confirmed by negative molecular biology and serological tests in another study [30]. The presence of a previous infection with an *Orthoflavivirus* in patients as well as the amplitude of anti‐ZIKV immunoglobulins (optical density ratio = OD (target)/OD (blank)) and seroneutralization titers were assessed as previously described [28]. Their kinetic characteristics, maximum amplitude, and the day when this amplitude was reached, were determined by extrapolation from experimental data (ODr and tiers), with a curve plotted using the Wood equation [31].

### 2.2 Cytokine Assay

A bead‐based multiplexed immunoassay was performed to measure the concentrations (pg/mL) of 14 cytokines (TNF‐α, IFN‐γ, IL‐10, IL‐12, IL‐17A, IL‐2, IL‐21, IL‐23, IL‐1β, IL‐5, IL‐4, IL‐6, GM‐CSF, IL‐13) using the MILLIPLEX map Kit HSTCMAG‐28SK (Millipore, Billerica, MA, USA), in accordance with the manufacturer's instructions. Each cytokine present in serum samples was captured by magnetic beads (BPLX MAG COOH, Luminex Inc.) coated with specific antibodies, then complexed with a biotinylated detection antibody and fluorescent streptavidin. Fluorescent signals were detected using the MAGPIX instrument and xPONENT software (Luminex Corp., Austin, TX, USA). Fluorescence intensity was converted to cytokine concentration using standard curves plotted with a 5‐parameter logistic model. For statistical analysis, samples whose fluorescence values could not be interpolated from the standard curve were replaced by half the minimum detection limit.

### 2.3 Statistical Analysis

The Kruskal–Wallis test with Dunn's post hoc test and the Benjamini–Hochberg procedure were used to compare the different time points and the control group. Correlation matrices (R Studio, v4.2.2) were constructed to relate these cytokine assay results to different variables previously described, such as IgG directed against ZIKV (Pearson), ZIKV microneutralization (Pearson), or duration of the symptoms (Spearman). Regarding the time‐dependent cytokine heatmap, the cytokine measurements were centered (mean subtracted) and scaled (by standard deviation). Each cytokine concentration was then summarized by its median value at each chosen time period. These transformed values were represented using a color code implemented with the heatmap R library. Cytokine hierarchical clustering relied on an agglomeration method of Euclidean distances between rows. Principal component analysis (PCA) was performed with the FactoMineR R package (scale unit = TRUE) and using the following variables: the age of the ZIKV‐infected patients, all cytokine measurements, the time points, and the presence or absence of a past *Orthoflavivirus* infection. The samples were visualized in PCA space using the factoextra package with PC1 and PC2 as coordinates.

## 3 Results

### 3.1 Serum Cytokine Kinetics During the First Month After Symptom Onset

**Figure 1:**
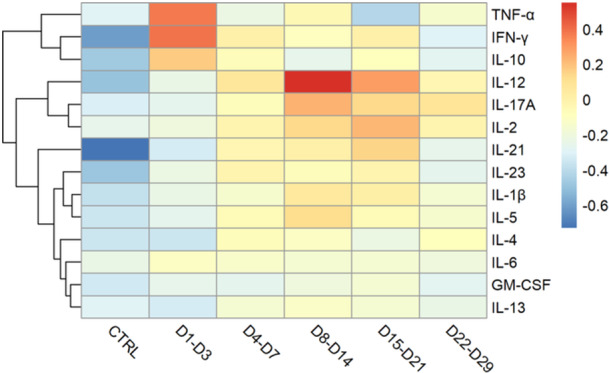
Heatmap visualizing the kinetics of normalized concentration of 14 cytokines (TNF‐α, IFN‐γ, IL‐10, IL‐12, IL‐17A, IL‐2, IL‐21, IL‐23, IL‐1β, IL‐5, IL‐4, IL‐6, GM‐CSF, IL‐13) in the serum from 36 patients infected with ZIKV up to 1 month after the onset of symptoms and ordered by hierarchical clustering. The color scale indicates the *z*‐score of the median concentration with red color for values above the median, blue below the median.

**Figure 2:**
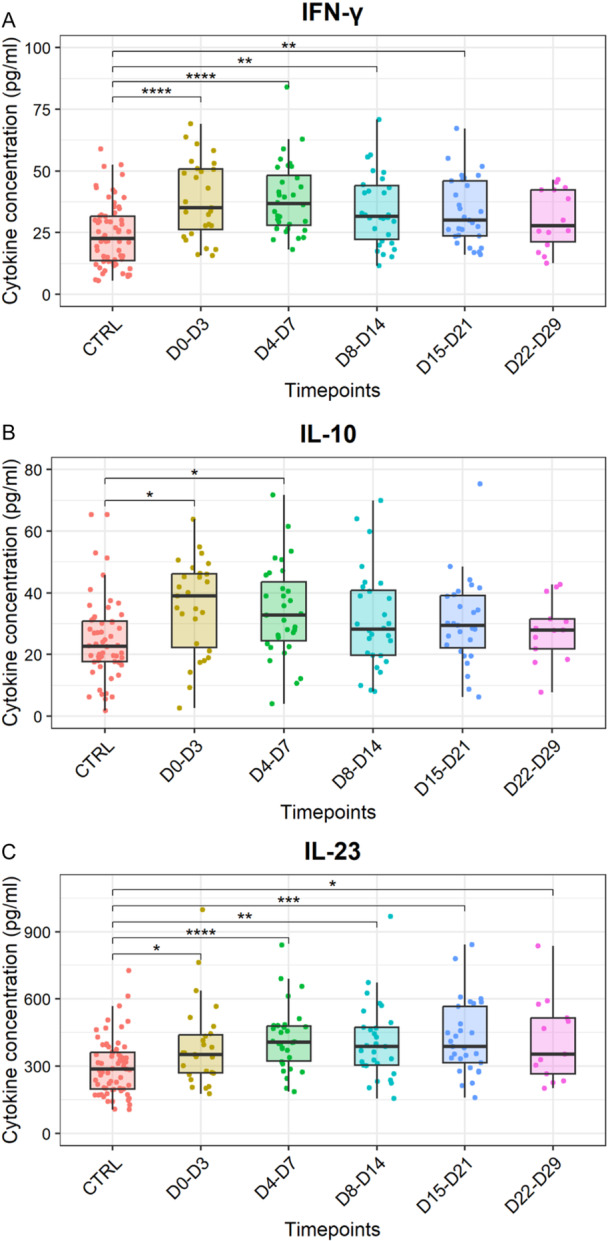
Kinetics of concentrations of three cytokines in the serum measured up to 1 month after the onset of symptoms. (A) Concentrations (pg/mL) of IFN‐γ in the serum from control individuals (not infected, CTRL, *n* = 67) and ZIKV‐infected patients (*n* = 36) at different days postonset of symptoms: Days 0–3 (D0–D3), Days 4–7 (D4–D7), Days 8–14 (D8–D14), Days 15–21 (D15–D21), and Days 22–29 (D22–D29). (B) Concentrations (pg/mL) of IL‐10. (C) Concentrations (pg/mL) of IL‐23. Asterisks indicate statistically significant differences between groups: *p < 0.05 **p < 0.01 ***p < 0.001 ****p < 0.0001 (Kruskal–Wallis test with Dunn's post hoc test and Benjamini–Hochberg correction).

The early cytokine response (from Days 0 to 3 after symptom onset) was dominated by the antiviral profile (Figures 1 and 2A, and Supporting Information S1: Figure 1). As the median incubation period for ZIKV infection was estimated at 6.8 days (95% CI [5.8–7.7 days]) [32], this early time point corresponds to 6–11 days after infection. Hierarchical clustering of the response kinetics associated the early elevation of IFN‐γ with TNF‐α and IL‐10 (Figures 1 and 2A,B). During the second week postonset of symptoms, the Th1–Th17 response was dominant, particularly IL‐12 and IL‐17A (Figure 1 and Supporting Information S1: Figure 1). Three weeks after the onset of symptoms (Days 15–21), a peak in IL‐2 and IL‐21 was observed (Figure 1 and Supporting Information S1: Figure 1). Four weeks after symptom onset, IL‐23 still showed a statistically significant difference compared to the control group (*p* < 0.01, Kruskal–Wallis test with Dunn's post hoc test and Benjamini–Hochberg correction), while all the other cytokines had returned to homeostasis (Figure 2C and Supporting Information S1: Figure 1). IL‐23 did not peak, but increased steadily up to 1 month in ZIKV‐infected individuals in the cohort (Figure 2C and Table 1). The presence of a previous infection with an *Orthoflavivirus* in patients has no impact on the cytokine profile, as illustrated by a PCA (Supporting Information S2: Figure 2). A Wilcoxon test was used to assess gender differences for each cytokine at each time point. No statistically significant differences were observed between male and female patients (Supporting Information S3: Supplementary File).

**Table 1:** Cytokine concentrations in patients infected with the Zika virus and in the control group during the first month after symptom onset.

	ZIKV‐infected patients (*n* = 36)					Control group (*n* = 67)
	D0–D3^a^	D4–D7	D8–D14	D15–D21	D22–D29	
GM‐CSF	108.1 (77.2, 135.5)	114.3 (92.2, 160.1)	112.6 (87.7, 160.4)	121.0 (99.7, 170.5)	106.3 (79.7, 128.2)	97.7 (75.8, 127.4)
IFN‐γ	35.2 (26.2, 52.1)	37.2 (27.6, 51.7)	32.1 (21.7, 46.9)	30.7 (23.7, 46.7)	30.1 (20.1, 42.4)	22.6 (13.4, 31.7)
TNF‐α	8.5 (6.4, 11.7)	7.4 (5.8, 9.6)	7.1 (5.8, 9.2)	7.5 (6.1, 8.8)	7.5 (6.1, 8.3)	7.2 (5.8, 9.3)
IL‐1β	3.8 (3.0, 4.4)	4.3 (3.3, 5.7)	4.6 (3.6, 6.1)	4.8 (4.0, 6.6)	4.4 (3.3, 5.0)	3.3 (2.6, 4.4)
IL‐2	7.7 (6.1, 9.4)	9.0 (8.2, 11.0)	10.0 (7.7, 12.1)	10.5 (7.8, 12.6)	9.1 (7.6, 11.0)	7.6 (3.6, 9.5)
IL‐4	33.6 (25.2, 53.2)	46.6 (33.9, 60.0)	42.9 (33.5, 63.6)	41.4 (32.2, 57.5)	45.1 (35.3, 59.6)	31.5 (21.6, 46.3)
IL‐5	8.7 (5.9, 11.9)	11.2 (8.2, 14.0)	12.3 (7.5, 13.7)	11.0 (8.0, 14.5)	10.4 (6.9, 11.8)	7.8 (4.6, 10.2)
IL‐6	3.1 (2.4, 4.2)	3.0 (2.4, 3.7)	2.8 (2.1, 3.9)	2.9 (2.5, 3.7)	2.5 (2.4, 3.0)	2.4 (1.6, 3.8)
IL‐10	39.0 (22.3, 47.3)	33.1 (24.6, 46.9)	29.2 (19.8, 43.2)	30.1 (22.6, 41.8)	27.9 (21.0, 33.7)	23.0 (17.8, 32.2)
IL‐12	6.6 (5.6, 9.0)	8.5 (6.2, 10.1)	8.4 (6.1, 10.1)	8.3 (6.3, 9.9)	7.6 (6.3, 8.5)	5.8 (3.6, 8.0)
IL‐13	5.2 (3.5, 10.9)	8.1 (5.4, 10.7)	7.9 (5.5, 11.0)	7.3 (5.7, 10.9)	7.0 (5.1, 9.7)	5.9 (4.4, 8.5)
IL‐17A	15.7 (10.2, 23.3)	19.0 (14.0, 27.8)	23.7 (15.3, 26.3)	23.2 (15.6, 29.8)	23.0 (15.7, 23.9)	14.3 (5.6, 20.6)
IL‐21	318.3 (243.5, 533.9)	442.2 (332.6, 578.4)	430.8 (339.2, 666.7)	469.9 (320.2, 788.1)	350.4 (265.9, 585.9)	227.6 (149.1, 431.1)
IL‐23	351.9 (270.2, 461.1)	410.5 (328.7, 498.3)	392.3 (304.8, 545.1)	399.6 (310.3, 581.4)	466.8 (266.3, 576.2)	287.6 (196.2, 363.8)

*Note:* All cytokines concentrations are in pg/mL.

^a^ D, days after symptom onset, median and (25th percentile, 75th percentile).

### 3.2 Correlation Between Circulating Cytokine Concentrations and Serological Outcomes

**Figure 3:**
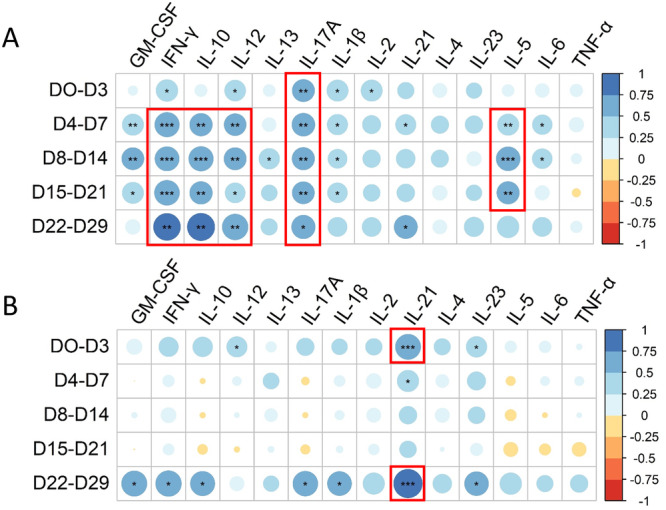
Correlation between circulating cytokine concentrations and serological outcomes. (A) Correlation matrix (Pearson) between the 14 cytokine concentrations and the maximum amplitude of the total ZIKV IgG response (optical density—OD, ELISA) at different time points (days—D) postonset of symptoms. (B) Correlation matrix (Pearson) between the 14 cytokine concentrations and the maximal seroneutralization titer at different time points (days—D) postonset of symptoms. The color and size of the dots (scale next to graph) indicate the degree of correlation (Pearson, *R* ^2^) between the cytokine concentrations and the serological parameters (small to large indicating low to high correlation).

Correlations between cytokine concentrations and previously published serological data from the same patients infected with ZIKV [28] were investigated. The maximum amplitude of the anti‐ZIKV IgG response (median value of eight (ODr), Level_max_ values in Table 1 in Marquine et al. [28]) was positively correlated with cytokine concentrations, mainly IFN‐γ, IL‐10, IL‐12, IL‐17A, and IL‐5 (Figure 3A). At an early stage (from Days 0 to 3 after symptom onset), levels of IFN‐γ (*R* ^2^ = 0.49, *p* = 0.01, Pearson), IL‐12 (*R* ^2^ = 0.46, *p* = 0.02, Pearson), IL‐1β (*R* ^2^ = 0.47, *p* = 0.02, Pearson), IL‐2 (*R* ^2^ = 0.47, *p* = 0.02, Pearson), and IL‐17A (*R* ^2^ = 0.52, *p* = 0.006, Pearson) were statistically significantly correlated with the magnitude of the anti‐ZIKV IgG response (Figure 3A). With regard to maximum seroneutralization titers (median value of 151, Level_max_ values in Table 1 in Marquine et al. [28]), a statistically significant positive correlation was observed with IL‐21 in the early stage (Days 0–3) (*R* ^2^ = 0.66, *p* < 10^−3^, Pearson) and late stage (> 21 days) (*R* ^2^ = 0.84, *p* < 10^−3^, Pearson) (Figure 3B).

### 3.3 Correlation Between Circulating Cytokine Concentrations and Clinical Outcomes

**Figure 4:**
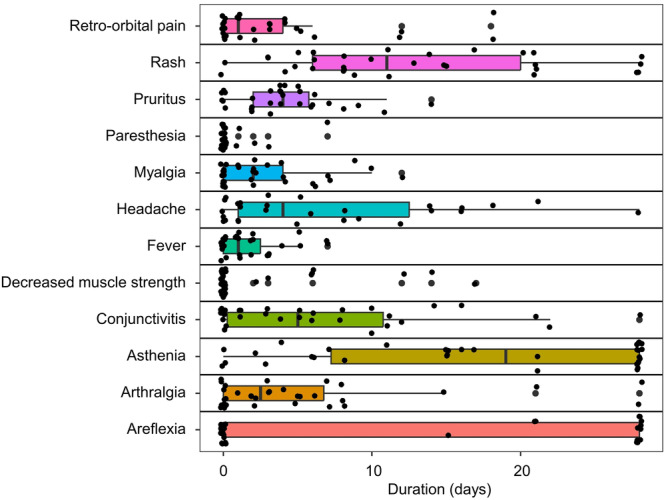
Boxplot (median, IQR, and 95% CI) representing the duration of main clinical symptoms observed in the cohort during the first month postonset. Each dot represents one patient (*n* = 34).

**Figure 5:**
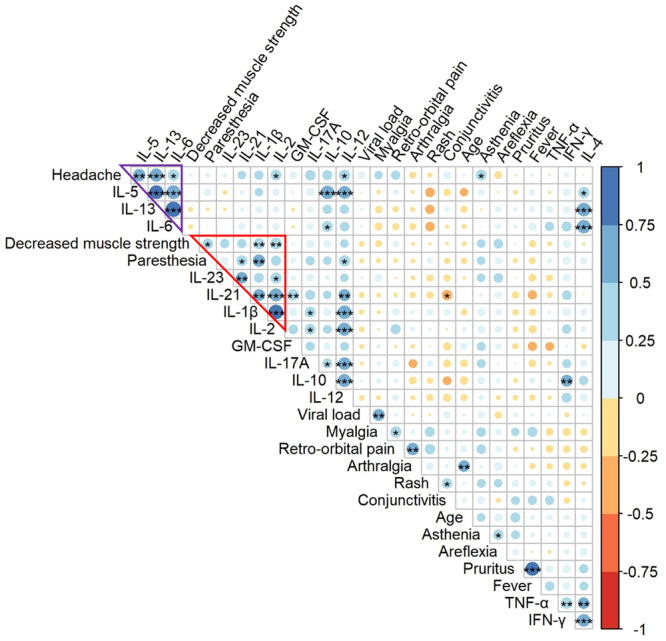
Correlation matrix (Spearman) between the 14 cytokine concentrations at an early time point (Days 0–3 postonset of symptoms) and the duration of clinical symptoms (first month postonset). The red triangle surrounds the correlation between early Th1–Th17 cytokine response and the duration of neurological symptoms (paresthesia and decreased muscle strength). The purple triangle surrounds the correlation between early Th2 cytokine response and the duration of headache. The color and size of the dots (scale next to graph) indicate the degree of correlation (Spearman, *ρ*) between the parameters (small to large indicating low to high correlation). Asterisks indicate statistically significant differences of the correlations: *p < 0.05, **p < 0.01, ***p < 0.001.

In the current cohort, the median age was 40 years [IQR 34–45] and the majority of patients were male (71%). ZIKV infections were mild to moderate, with the main symptoms being rash (92%), asthenia (73%), and headache (69%). No patient developed severe neurological symptoms, but 51% experienced areflexia during the first month after symptom onset, and 20% complained of decreased muscle strength and paresthesia [1] for 2–17 days (min–max) and 1–7 days, respectively (Figure 4). These durations were statistically significantly and positively correlated with early concentrations of IL‐1β (*ρ* = 0.56, *p* = 0.003, Spearman), IL‐2 (*ρ* = 0.49, *p* = 0.007, Spearman), and IL‐21 (*ρ* = 0.44, *p* = 0.03, Spearman) (Figure 5), and more generally with the Th1–Th17 response, whereas an early elevation of Th2 cytokines (IL‐5, IL‐13) was statistically significantly and positively correlated with headache duration (*ρ* = 0.57, *p* = 0.003 and *ρ* = 0.65, *p* < 0.001, respectively, Spearman) (Figure 5). Age was positively correlated with the duration of arthralgia (*ρ* = 0.51, *p* = 0.004) and initial viral load with myalgia (*ρ* = 0.53, *p* = 0.003), but no link was established with neurological disorders or cytokine concentration.

## 4 Discussion

In the present study, serum cytokine kinetics during the first month after symptom onset were performed in 36 ZIKV‐infected patients with mild to moderate symptoms. The early cytokine response was dominated by an antiviral profile characterized by elevated levels of IFN‐γ and TNF‐α, which act synergistically to promote antiviral functions [33], as well as IL‐10, a key regulator of excessive inflammation. The latter mainly displays anti‐inflammatory and resolutive properties protecting the host against tissue damage during the acute phase of infection [34]. Previous cohorts have also reported an increase in serum IL‐10 levels during the early phase of ZIKV infection [20, 25]. However, some deleterious effects of dysregulated IL‐10 have been described, such as the promotion of autoimmunity [35] and elevated serum levels have been observed in patients with severe Guillain–Barré Syndrome [36]. During the second week after symptom onset, the Th1–Th17 response was dominant, particularly IL‐12 and IL‐17A. IL‐12 may have different roles, ranging from induction of Th1‐T cell responses to enhancement of NK and CD8^+^ T cells cytotoxicity. IL‐17A induces Th17 polarized responses, whose antiviral functions can be extended: Th1‐response enhancement, activation and survival of cytotoxic CD8^+^ T cells, as observed during West Nile virus infection [37], and protective inflammatory response with low killing activity of cytotoxic CD8^+^ T cells [38]. But IL‐17 can also promote viral infections and tissue damage [39]. Its excessive activation could therefore contribute to the pathophysiology of Guillain–Barré syndrome [40], a fairly common neurological complication associated with ZIKV infection [3, 6]. Furthermore, this severe peripheral neuropathy appears much earlier following the onset of ZIKA disease symptoms than after infection with other arboviruses such as Chikungunya [5]. In a case‐control study of a ZIKV‐associated Guillain–Barré syndrome outbreak in French Polynesia [6], the neurological complication appeared 6 days [IQR 4–7] after symptom onset. This corresponds to the increase in the Th17 immune response observed in our follow‐up cohort. However, other immune mediators seem to be linked to the development of severe neurological complications [21, 41], which could differ since only nonsevere clinical manifestations were observed during the 1‐year follow‐up.

Three weeks after the onset of symptoms, a peak in IL‐2 and IL‐21 was observed. IL‐2 is a pleiotropic cytokine involved in pro‐ and anti‐inflammatory T cell differentiation and homeostasis [42], while IL‐21 is produced by NK T cells, CD4^+^ Th17 T cells, and, more potently, follicular helper T (Tfh) cells, which promote germinal center formation, B cell differentiation, and immunoglobulin production [43]. A higher level of serum IL‐21 was also observed in another cohort of ZIKV‐infected patients after the acute phase of the disease [25], as well as during primary or secondary dengue infections [44]. Four weeks after the onset of symptoms, IL‐23, a key cytokine for the maintenance and expansion of Th17 cell, was consistently increased for up to 1 month in ZIKV‐infected individuals in the cohort compared to the control group.

Associations between circulating cytokine concentrations and serological outcomes at the early stage of infection were evaluated. IL‐17A was positively correlated with the maximum amplitude of anti‐ZIKV IgG response, and IL‐21 was positively correlated with the maximum seroneutralization titers, both in the early and late stage of infection. IL‐17A and IL‐21 are both markers of Th17 response and their early presence may be critical for the B cell response, especially IL‐21 at an early stage, which may sustain germinal center and B cell maturation [43]. It could be hypothesized that individuals with higher seroneutralization titers may have prolonged germinal center reaction, with persistence of IL‐21 at a later stage following infection.

Relationships between circulating cytokine concentrations and clinical outcomes of patients infected with ZIKV were explored. The duration of decreased muscle strength and paresthesia was positively correlated with the Th1–Th17 responses (IL12, IL‐1β, IL‐2, IL‐23, and IL‐21), while the duration of headaches was correlated with an early elevation of Th2 cytokines (IL‐5, IL‐13). None of them correlated with viral load, and may rely on an excessive immune response. Other studies [25, 27] have also reported a positive correlation between IL‐5 levels and headaches during ZIKV infection. In contrast, we did not observe any association between high IL‐10 levels and arthritis [27] or myalgia [25] in the present study. Nevertheless, the duration of these symptoms correlated with age and viral load in our study, suggesting that they were driven by viral activity. The positive correlation of Th17 cytokines with the magnitude of the humoral response, as well as the duration of neurological symptoms suggests a bivalent role of Th17 response in ZIKV pathophysiology, as it may induce an excessive immune response. Antigen mimicry, bystander activation, viral neurotropism, and cytotoxicity have been hypothesized as mechanisms underlying the development of autoimmune neurological conditions associated with ZIKV infection [45]. In a recent mouse model of inflammatory neuropathy, a high accumulation of CD4^+^ T cells that secrete IL‐21 was observed in peripheral nerves. A combination of techniques (single‐cell RNA sequencing, histology, and cytometry with intracellular cytokine staining) was used to characterize these cells as peripheral Tfh‐like cells, some of which also secreted IFN‐γ and IL‐10. Knocking down the IL21 receptor protected the animals developing. This was associated with a decrease in the infiltration of pathological CD4^+^ T cells, as well as a reduction in the number of innate cells, which are also involved in this process [46]. In humans, the number of circulating Tfh cells increased in a subgroup of patients with Guillain–Barré syndrome [47]. Though, IL21 is critical for the development of Tfh cells [48] and for the maturation and differentiation of B cells. A positive correlation has also been observed between elevated serum levels of IL‐21 and antibody levels in patients infected with the dengue virus [44]. However, the regulation of the fate of B cells is complex since IL‐21 could also induce the apoptosis of naïve B cells [49]. An important limit of our study is the absence of cellular data to refine the origin of the circulating cytokines and the functional status of cell subtypes, as not only time but location and origin matter. In a pediatric cohort of ZIKV‐infected children, PBMC phenotyping using CyTOF identified CD14^+^ monocytes expressing CD169 as key factors of the immune response, as well as CXCL10 upregulation. In this study, there was no impact of prior *Orthoflavivirus* infection on the innate immune response to ZIKV, in accordance with our data obtained in an adult population [50]. Another CyTOF characterization of PBMC, isolated from viremic adults in endemic areas (blood donors followed over 3 months), revealed a coordinated immune cellular signature associated with higher titers of ZIKV‐neutralizing antibodies. During the acute phase of ZIKV infection, the number of specific innate and adaptive cell types temporarily increased, including intermediate CD14^+^CD16^+^ monocytes, CD69 + NK, HLA‐DR^+^CD38^+^ non‐naïve CD8^+^ T cells, Th1 CD4^+^ T cells, and Tbet^+^ plasma cells [51]. Unfortunately, this study neither assessed circulating Tfh cell subpopulations very precisely nor included cytokine measurements (in serum or intracellularly), that would have helped to interpret the origin of circulating IL‐21 and other Th17 cytokines.

Overall, this longitudinal cytokine monitoring provides important chronological information on the immune responses to mild‐to‐moderate ZIKV infection: (i) an early antiviral response dominated by IFN‐γ and TNF‐α, controlled by an early increase in the immune modulator IL‐10; (ii) a Th‐1 response that peaks between 1 and 2 weeks after symptom onset, followed by a Th17 response that peaks 1 week later; (iii) resolution of the Th1 response by the third week, but persistence of the Th17 response for up to a month. This response pattern did not differ with regard to dengue serological status. Finally, the early increase in Th17 cytokines was positively correlated with maximal IgG response and seroneutralization titers, which may have a beneficial effect on infection, but was also correlated with the duration of neurological symptoms and may therefore have deleterious effects for the patient. Given that Guillain–Barré syndrome has been associated with ZIKV infection in many countries, and that the maternal Th1/Th17 profile after ZIKV infection has been implicated in congenital Zika syndrome in Brazil [13], the balance and early increase of the Th17 response to ZIKV should be considered of the greatest importance. Further research is still needed to better understand its precise role in the pathogenesis of long‐term symptoms following ZIKV infection and to identify specific immune signatures that could predict the clinical outcome of the disease.

## Author Contributions

Conceptualization: Marie Mura and Sébastien Briolant. Methodology: Solène Marquine, Marie Mura, Gilda Grard, Cyril Badaut, and Sébastien Briolant. Software: Solène Marquine and Marie Mura. Formal analysis: Solène Marquine, Aurélie Trignol, and Marie Mura. Resources: Cyril Badaut and Sébastien Briolant. Writing: Solène Marquine, Marie Mura, and Aurélie Trignol. Visualization: Solène Marquine and Marie Mura. Supervision: Aurélie Trignol, Marie Mura, Gilda Grard, Cyril Badaut, and Sébastien Briolant. All authors have read, edited, and agreed to the published version of the manuscript.

## Ethics Statement

Ethical approval was given by the Comité de Protection des Personnes Sud Méditerranée I for the “Etude descriptive prospective de la maladie à virus Zika au sein de la communauté de défense des Forces Armées en Guyane ZIFAG” and was registered on February 29, 2016, as RCB: 2016‐A00394‐47. All research was performed in accordance with relevant guidelines from the Declaration of Helsinki.

## Consent

All necessary patient informed written consent was obtained, and the appropriate institutional forms were archived.

## Conflicts of Interest

The authors declare no conflicts of interest.

## Supporting information


**Figurementary Figure 1:** Kinetics of concentrations (pg/mL) of eleven cytokines in the serum measured in the serum from control individuals (not infected, CTRL, *n* = 67) and ZIKV‐infected patients (*n* = 36) at different days postonset of symptoms: Days 0–3 (D0–D3), Days 4–7 (D4–D7), Days 8–14 (D8–D14), Days 15–21 (D15–D21), and Days 22–29 (D22–D29). Asterisks indicate statistically significant differences between groups: *p < 0.05, **p < 0.01, ***p < 0.001 (Kruskal–Wallis test with Dunn's post hoc test and Benjamini–Hochberg correction).

**Supporting Figure 2:** Principal component analysis (PCA) representation of all the cytokines measured at different timepoints in the peripheral blood of ZIKV‐infected patients (*n* = 36). Individuals are grouped according to past infection with *Orthoflavivirus*, categorized as previously exposed (Yes) and unexposed (No), with 95% confidence ellipses for each group.

Supporting File: Wilcoxon test comparing cytokine concentrations between male and female patients at each timepoint. No statistically significant differences were observed after Benjamini–Hochberg correction.

## Data Availability

The data are contained within the manuscript. The analysis script in R can be made available upon request from the corresponding author.
