# Supplementary figures and images for: Longitudinal Monitoring of Systemic Cytokines After Mild Zika Virus Infection Revealed an Association Between Th17 Polarization and Clinical and Serological Outcomes
Source: J Med Virol. 2026 Jan 20;98(1):e70813. doi: 10.1002/jmv.70813 (PMC12817652; doi:10.1002/jmv.70813)

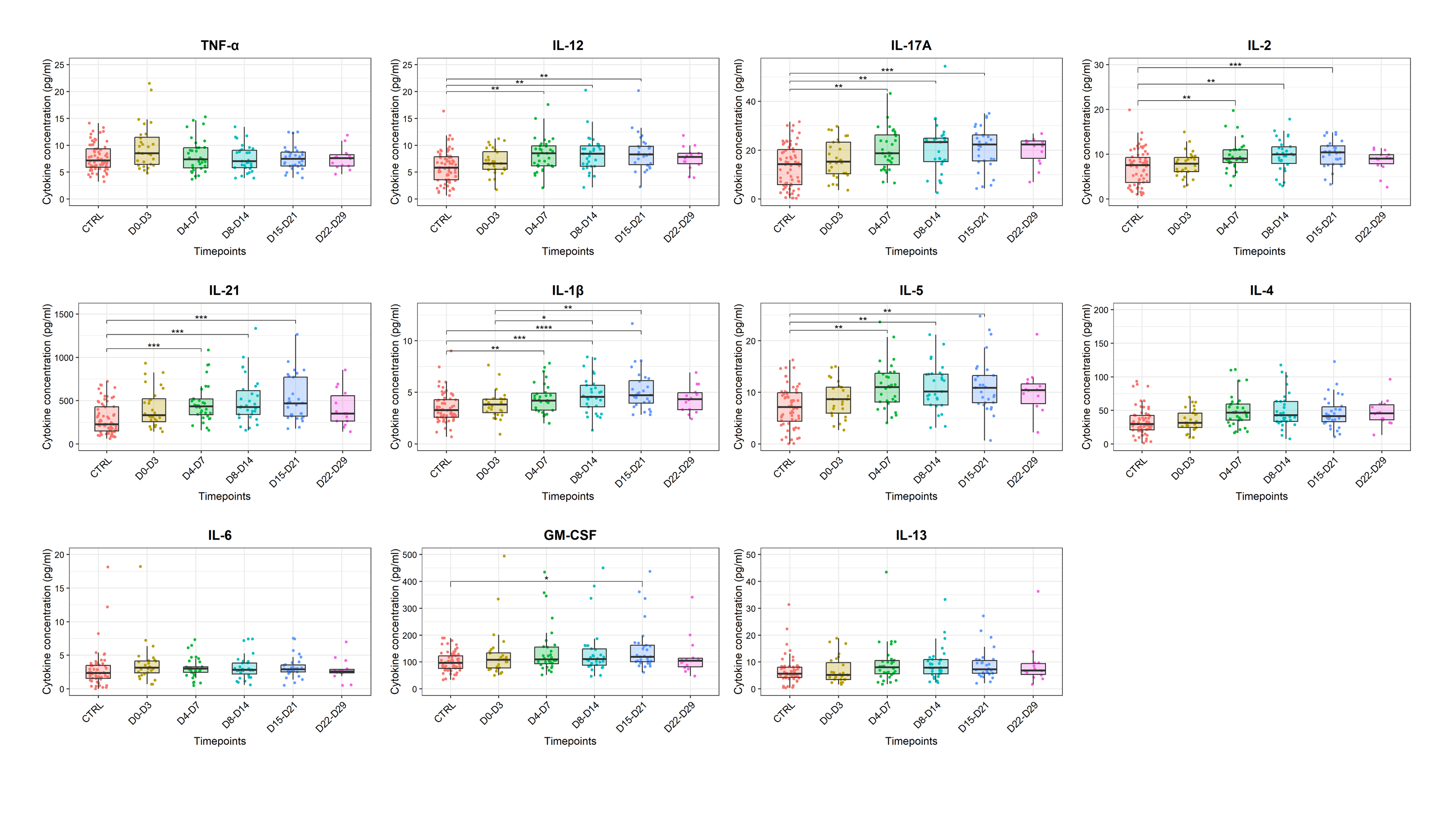

Supplement: Supplementary file 1 — Figurementary Figure 1: Kinetics of concentrations (pg/mL) of eleven cytokines in the serum measured in the serum from control individuals (not infected, CTRL, n = 67) and ZIKV‐infected patients (n = 36) at different days postonset of symptoms: Days 0–3 (D0–D3), Days 4–7 (D4–D7), Days 8–14 (D8–D14), Days 15–21 (D15–D21), and Days 22–29 (D22–D29). Asterisks indicate statistically significant differences between groups: *p < 0.05, **p < 0.01, ***p < 0.001 (Kruskal–Wallis test with Dunn's post hoc test and Benjamini–Hochberg correction). [file JMV-98-e70813-s002.tif]

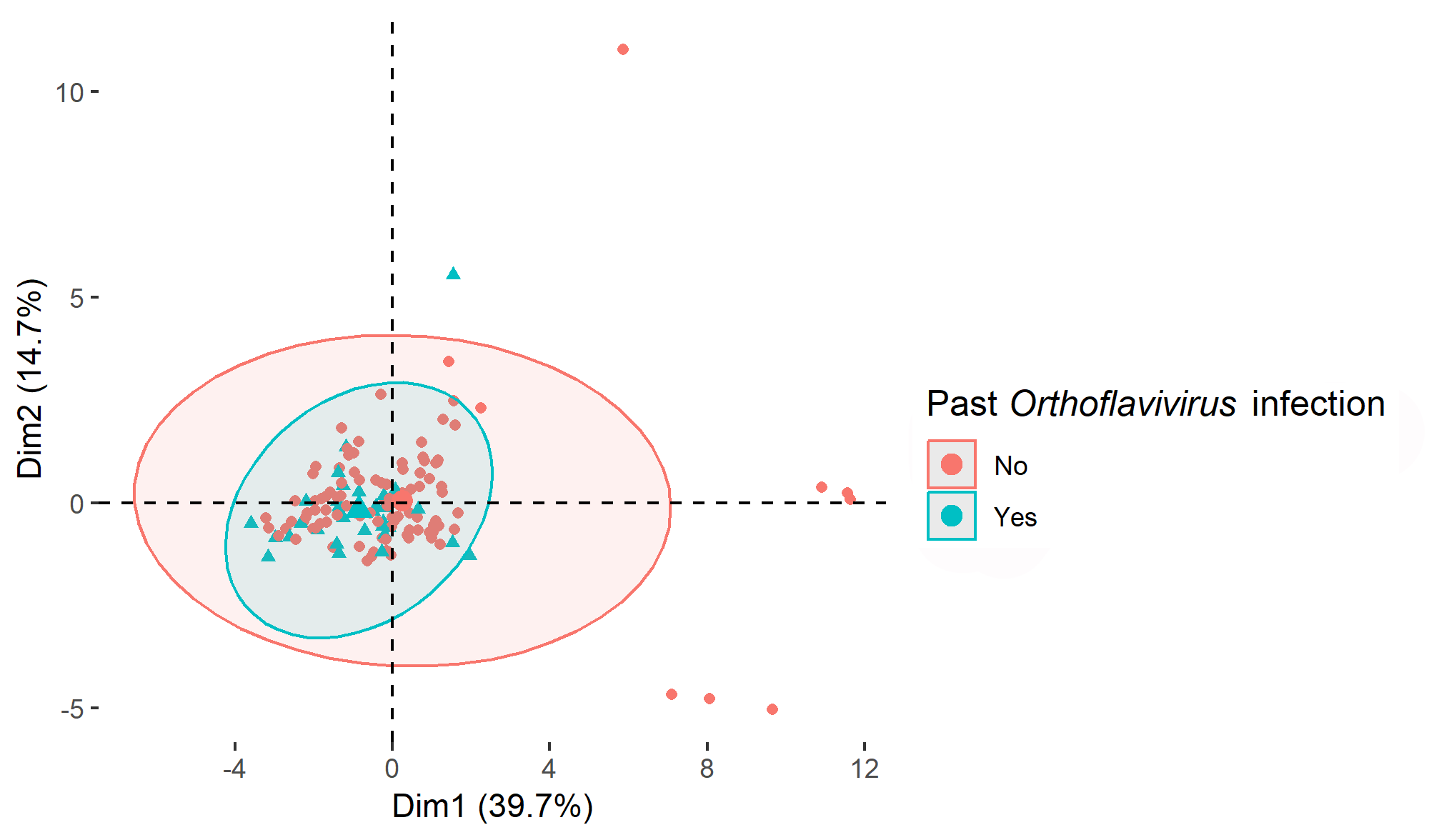

Supplement: Supplementary file 2 — Supporting Figure 2: Principal component analysis (PCA) representation of all the cytokines measured at different timepoints in the peripheral blood of ZIKV‐infected patients (n = 36). Individuals are grouped according to past infection with Orthoflavivirus, categorized as previously exposed (Yes) and unexposed (No), with 95% confidence ellipses for each group. [file JMV-98-e70813-s003.tif]
